# Treatment of Roots

**Published:** 1868-10

**Authors:** 


### TREATMENT OF ROOTS.

THE almost universal practice of extraction of the roots of all teeth, the crowns of which have become so much decayed, as not to be susceptible of filling, is one which it may not be amiss to consider a little. The indications for the extraction of teeth are not very definite or uniform, a condition that would be to one operator a clear indication for extraction might be quite otherwise to another. This variation of indications, is, however, wholly dependent upon the variation in both the natural and acquired abilities, of different operators.

The point, however, to which we will especially direct attention is the retention of the healthy roots of teeth, in the jaws—roots free from disease, or that may by proper treatment become so. A solid root in the alveolus, is no more liable to become the occasion of disease, than before its pulpless crown was destroyed. It is true that roots decayed to a mere conical shell, will occasion irritation and frequently disease to some extent; but this plea is for those roots that are comparatively sound, without reference to the part of the mouth in which they are situated, and without reference to the insertion of artificial teeth. Several obvious advantages accrue from their preservation.

The alveolus, gums and other neighboring soft parts too, are retained with their natural form and position, in which, if the root was removed, a marked change rapidly occurs, marring symmetry and beauty, and perhaps vitiating enunciation. By the removal of a tooth, or its roots, the neighboring teeth loose support to some extent, they are liable to be, and usually are thrown out of their proper position, and that nicety of occlusion with the antagonizing teeth, so important in thorough mastication, is destroyed. How common is it to see such teeth, either failing altogether to make contact with their antagonist, or only touching them in a very inefficient manner, at a single point, edge or angle. So long as the alveolus is occupied by its complement of roots, such derangement can not occur. And, in addition to this, such roots usually render valuable service in mastication.

Every one is aware, at least who has had teeth extracted, that owing to sensitiveness of the parts operated upon, it is impossible to use the adjoining teeth, as before, and in the great majority of cases, they are used but little, or not at all, for a long time, and usually they are never afterward used as efficiently as before. The gum, even after the socket is thoroughly closed, makes but a very feeble resistance to many hard, rough substances that are brought in contact with it during mastication; hence, it is found that the teeth adjoining a vacant alveolus are, for a time, thrown out of use, at least so far as mastication is concerned, and necessarily suffer a want of exercise and cleanliness. The same is true, also, of their antagonists. Good firm roots, though dressed down even with the margin of the gums, will sustain the contact of rough hard substances equally as well as before the crown is lost, and protect the soft parts from injury—enabling contiguous teeth to be used as well as formerly.

Then, we would suggest, save all sound healthy roots, or those that can be made so, and especially if a plate is not to be inserted. If a portion of the crown of such tooth is standing which is of little or no value, cut it off, and dress down with the file almost or quite to the margin of the gum, and fill the canals of the roots, and any portion of pulp chamber that may remain after the dressing. If the pulp in such a tooth or root is living it of course should be removed, and the root or roots filled.

It is important in all such cases to dress down, if possible, till a smooth solid surface is obtained, one that will afford no lodgment for food or any foreign substance.

We have pursued this practice to some extent during the last fifteen years, and much more frequently during the last six years than previously, and have never in a single instance had occasion to regret it; we find, now, many cases of this kind that have been doing invaluable service for years; and it is by no means a small gratification to receive the thanks and expression of appreciation from grateful patients, who had presented themselves, anticipating the terrible operation of extraction.

Fellow practitioners try it and report. T.

